# A Two‐Photon Excitation Based Fluorogenic Probe for Sialome Imaging in Living Systems

**DOI:** 10.1002/advs.201500211

**Published:** 2015-09-02

**Authors:** Lei Rong, Chi Zhang, Qi Lei, Si‐Yong Qin, Jun Feng, Xian‐Zheng Zhang

**Affiliations:** ^1^ Key Laboratory of Biomedical Polymers of Ministry of Education and Department of ChemistryWuhan UniversityWuhan430072P.R. China; ^2^ State Key Laboratory of VirologyCollege of Life SciencesWuhan UniversityWuhan430072P.R.China; ^3^ School of Chemistry and Materials ScienceSouth‐Central University for NationalitiesWuhan430074P.R. China

**Keywords:** click reaction, fluorescence labeling, glycoengineering, zebrafish

## Abstract

**Taking advantage of the fluorescence property of alkyne‐modified naphthalimide,** the two‐photon excited fluorogenic probe Naph‐yne is used for azide‐tagged mannosyl glycoprotein imaging both at the cellular level and for vertebrate model organisms.

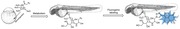

Sialic acid is prominently situated at the outer ends of the glycoproteins and glycolipids in every vertebrate cell membranes, which collectively comprise the sialome.^1^ This location advantage gives sialic acid the ability to mediate and/or modulate many cellular interactions emerged at the cell surface.^2^ For example, sialic acid could act as antirecognition agent and mask underlying recognition sites such as penultimate monosaccharides of glycan chains or antigenic proteins of cell membranes.^3^ Thus, in situ monitor of the sialic acid is of great importance and has attracted increasing attention in recent years.^4^ As one of the most commonly used approach, metabolic glycoengineering allows a certain sialic acid precursor to be incorporated into a cell‐surface glycan after a series of biosynthesis pathways, and ensure the further modification or fluorescent detection.

Compared to the commonly used detection techniques using fluorescent dyes, the recently emerged fluorogenic labeling method holds the merit of high signal‐to‐noise ratio, which is due to the off‐on fluorescence change of the probe before and after attached to the desired site, separately.^5^ This advantage make fluorogenic labeling a promising technique in many imaging conditions where it is not possible to wash away redundant fluorescent dyes due to the inherent adhesion and cellular internalization, such as real‐time imaging in living systems. During the past decade, various fluorogenic strategies have been exploited to fulfill different imaging needs, including in vivo labeling.^6^ Since azide and alkyne are the most commonly used functional groups in metabolic glycoengineering, azide–alkyne click reaction based fluorogenic probes were considered as promising tools for sialic acid imaging both in vitro and in vivo.^7^

We previously demonstrated that alkyne‐modified coumarin could be used as a fluorogenic probe for the sialylated glycans imaging in living cells through copper(I)‐catalyzed azide‐alkyne cycloaddition (CuAAC) reaction.^8^ Cells were pretreated with cell‐permeable, peracetylated *N*‐azidoacetylmannosamine (Ac_4_ManNAz), which could be converted to the corresponding azido‐tagged sialic acid and finally incorporated into sialomes on the cell surface. Owing to the merits of the CuAAC reaction, including high chemoselectivity, biocompatibility, fast reaction rate, and high yielding, the fluorogenic probe we used could efficiently imaging sialomes on cell surface owing to the formation of the 1,2,3‐triazole unit which could restore the fluorescent property of the coumarin via electron‐donating effects. Although this strategy enabled the in situ sialome imaging in living cells under no‐washing conditions, in vivo sialome imaging techniques were still of great importance for biosynthetic flux visualization. These techniques would provide an opportunity to monitor the dynamic changes in the sialome in a spatiotemporally resolved manner.

In this work, an alkyne‐functionalized fluorogenic probe (Naph‐yne) was employed for sialome imaging in living systems (**Figure** **1**). As a widely used fluorophore, naphthalimide could be modified with an alkynyl group substitution at the 4‐position and became a profluorophore with minimal fluorescence. After CuAAC reaction with the azido group and the formation of electron‐donating 1,2,3‐triazole unit, this profluorophore could change into a fluorescent probe with two‐photon excitation activity and high fluorescence quantum yielding. These properties provide Naph‐yne the opportunity to imaging azido‐containing sialomes in living systems.

**Figure 1:**
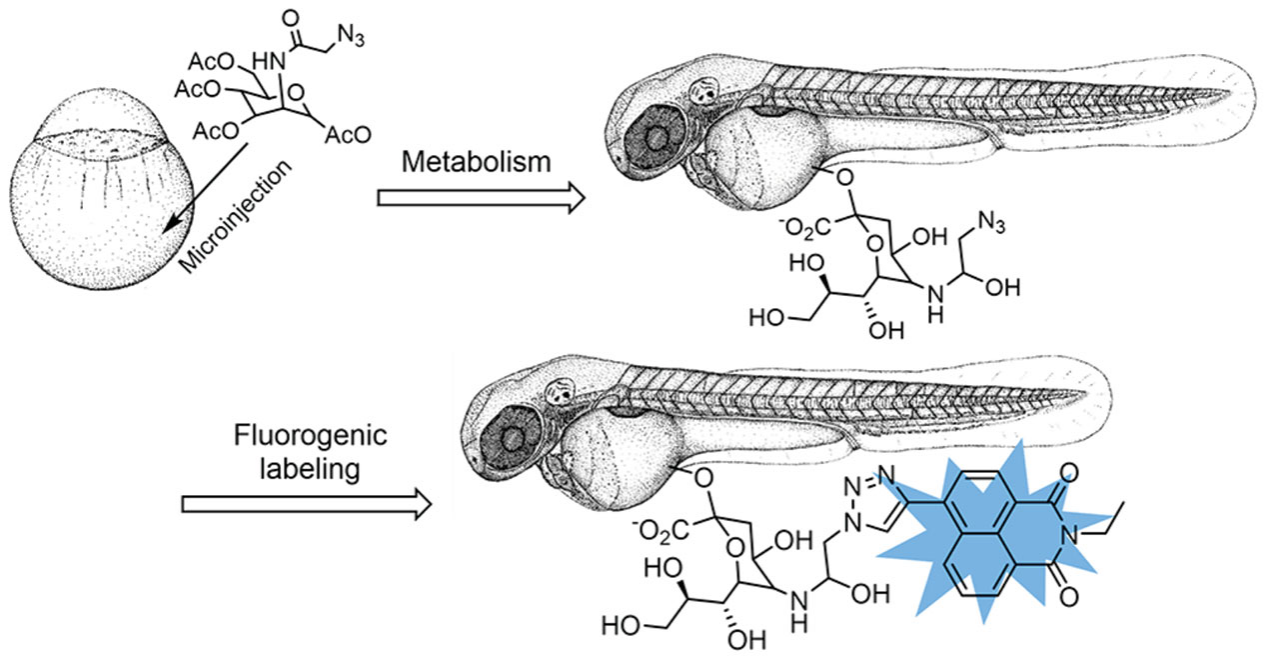
Scheme of azido sialome imaging in zebrafish using Naph‐yne. The Naph‐yne could react with the azido containing sialomes and result in the adduct with bright fluorescence.

The Naph‐yne was synthesized from 4‐bromo‐1,8‐naphthalic anhydride (Sigma‐Aldrich Co. LLC., USA), and confirmed by ^1^H NMR spectroscopy (see the Supporting Information). Reactivity and reaction‐activated fluorescent property of the Naph‐yne were characterized by performing a CuAAC reaction with Ac_4_ManNAz (Thermo Fisher Scientific Inc., USA) under simulated physiological conditions (37 °C in phosphate buffered saline (PBS, pH 7.4)) (**Figure** **2**A). Owing to the alkynyl group substitution at 4‐position, the fluorescence of the naphthalimide fluorophore could be efficiently quenched through the electron‐withdrawing effect (Figure 2B, dashed line). After CuAAC reaction, the electron‐donating property of the 1,2,3‐triazole unit ensures the adduct AN for producing a significant increase in fluorescence intensity (Figure 2B, solid line). The maximum emission of the AN was around 460 nm when excited at 370 nm. Moreover, the AN also demonstrated a reasonable two‐photon excitation activity. As shown in Figure 2C, the two‐photon excitation spectrum of AN in water exhibited a broadband from 690 to 830 nm and the maximum is around 740 nm, which is double of the optimum one‐photon excitation wavelength (370 nm). The AN excitation cross section (*δφ*) at 740 nm is around 25 GM, which is in agreement to the literatures.^9^, ^10^ In other words, the intrinsic brightness of the AN is sufficient for two‐photon microscopic applications. Moreover, the AN also exhibited a very high fluorescent quantum yield (0.73) in aqueous solution (see the Supporting Information for details).

**Figure 2:**
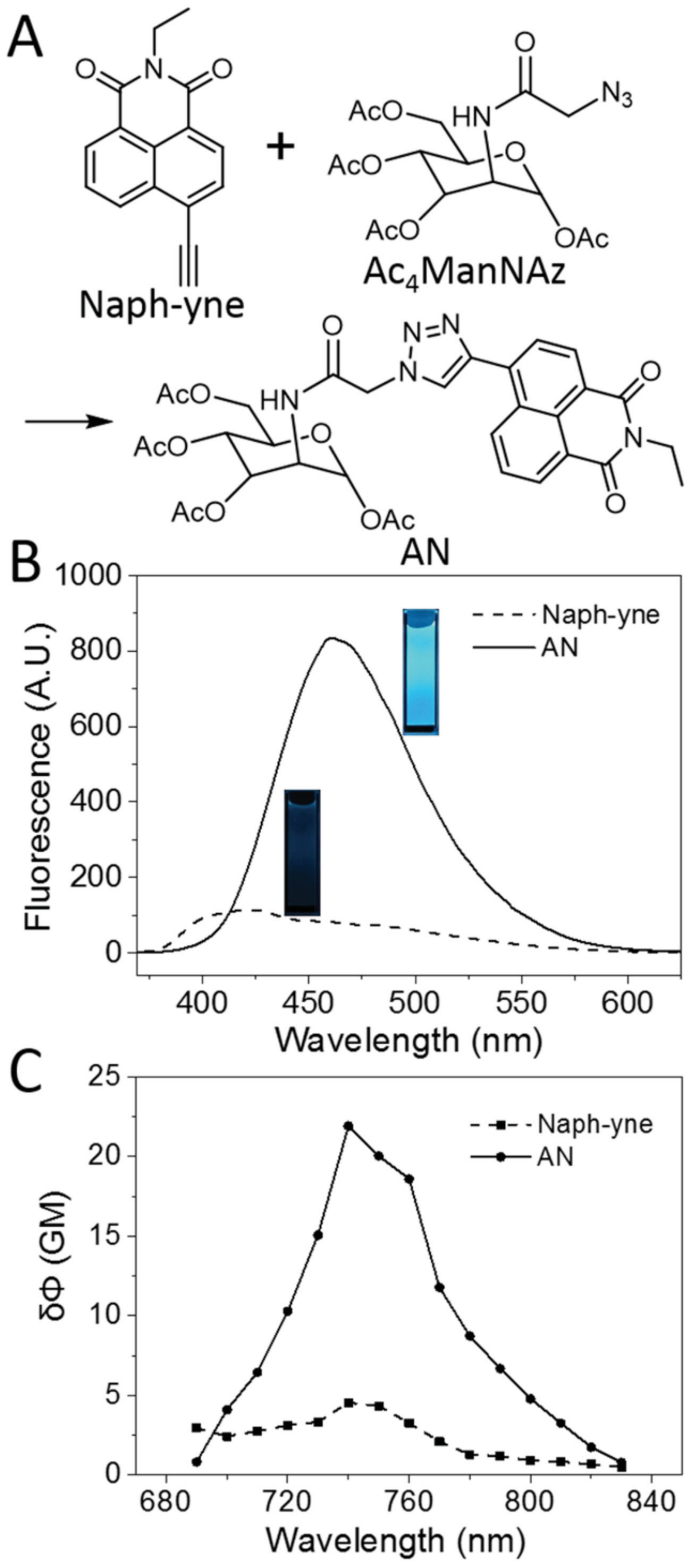
A) The fluorogenic CuAAC reaction of Naph‐yne and Ac_4_ManNAz results in a highly fluorescent adduct. B) Fluorescence spectra of Naph‐yne (5 × 10^−6^ m) and its adduct AN, insets: fluorescence emission photo of the Naph‐yne (left) and AN (right). C) Two‐photon excitation spectrum of 5 × 10^−6^ m Naph‐yne and 5 × 10^−6^ m AN in water. The estimated uncertainties for the two‐photon excitation cross‐section values (*δφ*) are ±15%.

The reaction rate between Naph‐yne and Ac_4_ManNAz in simulated physiological condition was investigated through monitoring fluorescent intensity of the reaction mixture at different time points. In the presence of the robust water‐soluble ligand, tris(3‐hydroxypropyltriazolylmethyl)amine (THPTA), the fluorescent intensity after CuAAC reaction increased sharply within 30 min (Figure S1, Supporting Information). In consideration of the cell compatibility,^11^ the reaction time used in subsequent experiments was restricted to 30 min.

Then, the Naph‐yne was used for the labeling of azide‐tagged mannosyl glycoproteins expressed on the cell surface. Human cervical adenocarcinoma (HeLa) cells were used as cellular model. Briefly, HeLa cells were treated with Ac_4_ManNAz (40 × 10^−6^ m) containing DMEM (Dulbecco's modified Eagle's medium, with 10% fetal bovine serum (FBS)) for 3 d to allow the azide groups be presented at the terminal position of sialomes by sialic acid pathway.^12^ The control cells (HeLa) were treated with d‐mannosamine (Sigma‐Aldrich Co. LLC., USA, 40 × 10^−6^ m) containing DMEM instead. After 3 d incubation, the medium was discarded and the cells were rinsed with PBS thrice to remove free Ac_4_ManNAz. After which, the cells were labeled with Naph‐yne using the same protocol based on our previous report.^8^ In an Eppendorf tube, CuSO_4_ and THPTA (2 and 10 equiv., respectively) were added to Naph‐yne (1 equiv.) containing PBS solution. Freshly prepared sodium ascorbate solution (10 equiv.) was added, and the labeling mixture was incubated at room temperature for 10 min before added to the cells. After 30 min incubation at 37 °C, the cells were fixed for cell nuclei stain using propidium iodide (PI, Sigma‐Aldrich Co. LLC., USA). Thereafter, the cells were detected by two‐photon excitation microscopy (Zeiss LSM 710, Carl Zeiss AG, Germany). As shown in **Figure** **3**, bright blue fluorescence was observed in the Ac_4_ManNAz pretreated cells while d‐mannosamine treated cells (control) showed no blue fluorescence signal. This bright blue fluorescence was ascribed to the successful cycloaddition of the alkynyl group in Naph‐yne with the azido group presented in sialomes, which came from the precursor Ac_4_ManNAz. Meanwhile, the colocalization of the PI and Naph‐yne showed that there was no overlap of azido‐tagged sialomes in cell nuclei. This observation was mainly due to the sialic acid biosynthesis pathways that do not associate with the cell nuclei.^13^ These results indicated that the Naph‐yne could be used as a sialomes probe in living cells.

**Figure 3:**
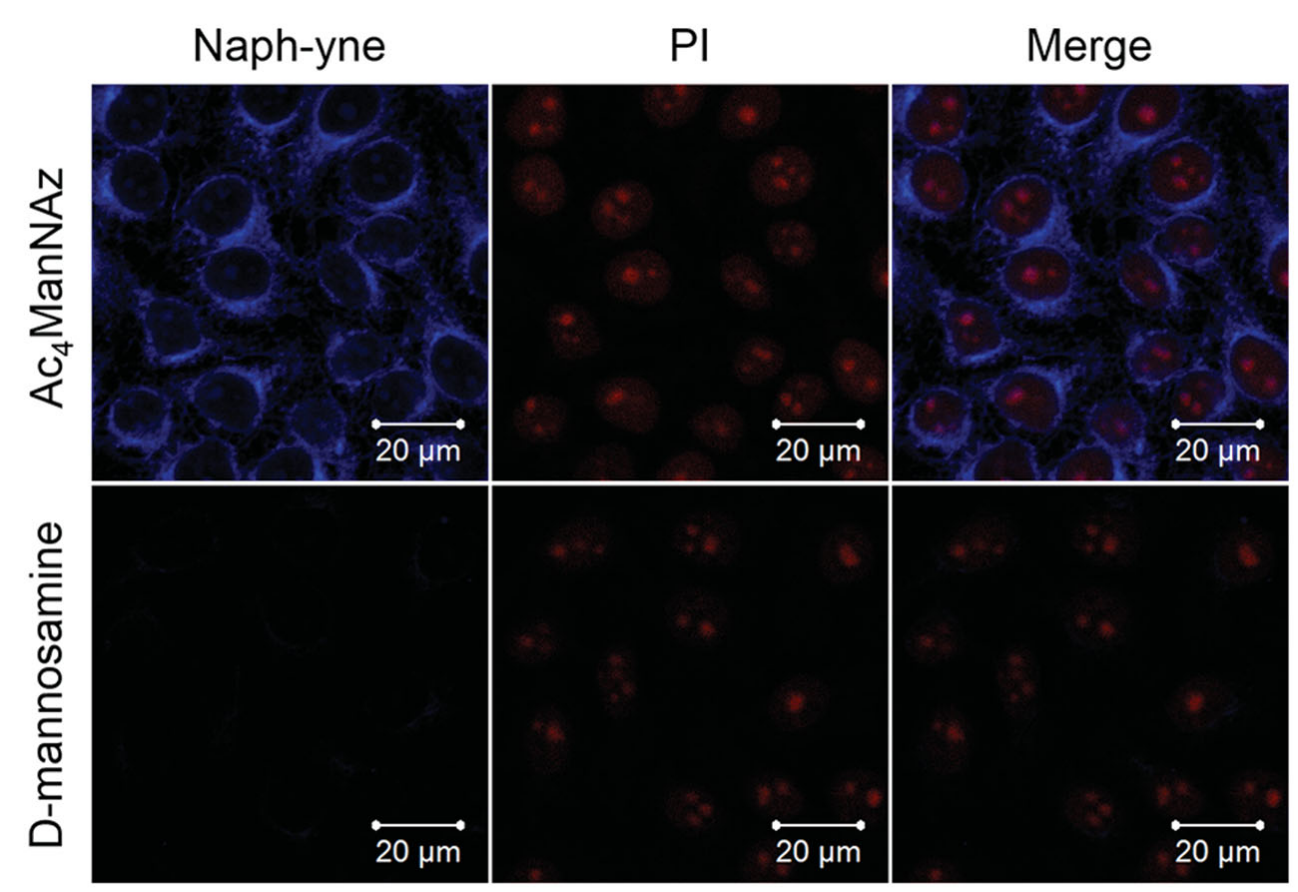
Fluorescence images of the Ac_4_ManNAz treated cells (top row) and d‐mannosamine treated cells (bottom row). The CuAAC adduct of Naph‐yne was showed in blue, and PI stained cell nuclei were in red. Scale bar: 20 μm.

Moreover, the cellular azide‐tagged mannosyl glycoproteins labeling efficiency of the Naph‐yne was also studied quantitatively using flow cytometry. HeLa cells were pretreated with Ac_4_ManNAz and labeled with Naph‐yne using the same protocol as described above. The percentage of fluorescent cells was measured using BD FACSAria III (BD Biosciences, USA). Ac_4_ManNAz pretreated cells without Naph‐yne labeling were presented as negative control. As shown in **Figure** **4**, over 99% Ac_4_ManNAz pretreated cells could be labeled using Naph‐yne. This indicated that, Naph‐yne could efficiently adduct to the azido groups presented on the outer side of the mannosyl glycoproteins, which was transformed from the Ac_4_ManNAz in cell culture medium.

**Figure 4:**
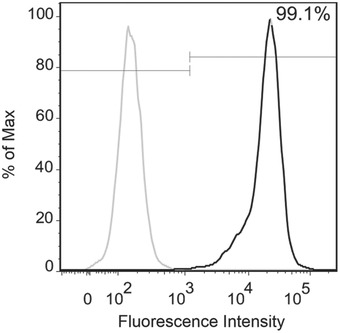
Quantitative measuring of the Naph‐yne labeled HeLa cells by flow cytometry. The black line refers to the cells labeled with Naph‐yne, and the gray line refers to the negative control.

Encouraged by the results above, the Naph‐yne was further employed for azide‐tagged mannosyl glycoproteins imaging in vivo. The zebrafish was chosen as model organism owing to its well‐investigated developmental program, ease of microinjection with exogenous reagents, as well as the transparent embryos.^14^, ^15^ Zebrafish embryos at the one‐cell stage were microinjected into the yolk with 1 nL of 50 × 10^−3^ m Ac_4_ManNAz.^16^ Embryos microinjected with 1 nL of 50 × 10^−3^ m d‐mannosamine were used as control. After microinjection, embryos were cultured at 28 °C using standard embryo culture protocol. When the embryos developed to 24 or 48 h postfertilization (hpf), they were labeled with Naph‐yne using the similar strategy as in cellular sialomes labeling described above. Briefly, at 24 or 48 hpf, the embryos were transferred into E3 medium containing CuSO_4_ (2 equiv.), THPTA (10 equiv.), Naph‐yne (1 equiv.), and sodium ascorbate (10 equiv.). The embryos were incubated at 28 °C for 30 min to ensure the successful labeling (see the Supporting Information for details). After which, the embryos were detected using two‐photon excitation microscopy (Zeiss LSM 710, Carl Zeiss AG, Germany). As shown in **Figure** **5**, the Ac_4_ManNAz‐microinjected embryos exhibited bright blue fluorescence in both 24 and 48 hpf while the control showed no visible fluorescent signal. This blue signal was also attributed to the successful cycloaddition of the alkynyl group in Naph‐yne with the azido group in the sialomes of the embryos. These azido groups were sourced from the microinjected Ac_4_ManNAz, which was converted to the corresponding azido‐tagged sialic acid and incorporated into sialomes through the biosynthetic pathways. In other words, the Naph‐yne could be used as an efficient fluorescent probe for azido‐tagged sialomes in zebrafish.^1^

**Figure 5:**
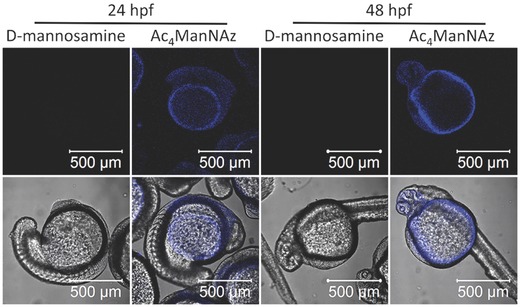
Fluorescent images of the Naph‐yne labeled zebrafish embryos at different time points after fertilization. (Top row) Fluorescent field of Naph‐yne labeled embryos; (bottom row) merged images of fluorescent field and bright field. Scale bar: 500 μm.

In summary, owing to the unique fluorescent properties of the Naph‐yne and its CuAAC reaction adduct, the Naph‐yne could be used as an efficient sialome imaging probe both in cellular level and for vertebrate model organism. Considering the reasonable two‐photon excitation activity and the high fluorescence quantum yield of the CuAAC adduct, we believe that the Naph‐yne could be further employed for the deeper tissue imaging in vivo.

## Experimental Section

*Cellular Experiments*: HeLa cells were acquired from China Center for Type Culture Collection (Wuhan, China). The cells were cultured in a humidified atmosphere containing 5% (v/v) CO_2_ at 37 °C using DMEM supplemented with 10% FBS, penicillin (100 units mL^−1^), and streptomycin (100 μg mL^−1^) (DMEM medium for short).

*Two‐Photon Excitation Imaging*: 3 d before imaging, the cells were placed on glass‐bottomed dished (Corning) using DMEM medium containing 40 × 10^−6^ m Ac_4_ManNAz. Cells used as control were cultured at the same condition using d‐mannosamine in place of Ac_4_ManNAz. After culturing for 3 d, the cells were rinsed thrice with PBS to remove excessive Ac_4_ManNAz or d‐mannosamine. Following which, PBS containing CuSO_4_ (2 equiv.), THPTA (10 equiv.), Naph‐yne (1 equiv.), and freshly prepared sodium ascorbate solution (10 equiv.) was added into the dishes after incubating in room temperature for 10 min. The cell‐containing dishes were incubated in 37 °C for 30 min and the cell nuclei were stained with PI using standard staining protocol. Then, the cells were imaged using LSM 710.

*Flow Cytometry*: HeLa cells were placed in six‐well cell culturing plate and pretreated with Ac_4_ManNAz containing DMEM medium 3 d before Naph‐yne labeling. The cells were labeled with Naph‐yne using the same protocol as described above. And Ac_4_ManNAz pretreated cells without Naph‐yne labeling were used as negative control. The cells were dissociated with 20% Trypsin, collected, washed with PBS thrice, and resuspended in PBS. The flow cytometry data were collected using BD FACSAria III.

*Zebrafish Fluorescence Imaging*: Zebrafish was acquired from China Zebrafish Resource Center (CZRC) (Wuhan, China).

Zebrafish eggs were fertilized and the Ac_4_ManNAz were microinjected into the yolk after successful fertilization. d‐mannosamine microinjected embryos were used as control. The embryos were cultured in E3 medium at 28 °C. At 24 or 48 hpf, the embryos were transferred into E3 medium containing CuSO_4_ (2 equiv.), THPTA (10 equiv.), Naph‐yne (1 equiv.), and freshly prepared sodium ascorbate solution (10 equiv.), and incubated at 28 °C for 30 min. After which, the embryos were transferred into fresh E3 medium and imaged using LSM 710.

## Supporting information

As a service to our authors and readers, this journal provides supporting information supplied by the authors. Such materials are peer reviewed and may be re‐organized for online delivery, but are not copy‐edited or typeset. Technical support issues arising from supporting information (other than missing files) should be addressed to the authors.

Supplementary
