# Supplementary material for: A Two‐Photon Excitation Based Fluorogenic Probe for Sialome Imaging in Living Systems
Source: Adv Sci (Weinh). 2015 Sep 2;3(1):1500211. doi: 10.1002/advs.201500211 (PMC5049655; doi:10.1002/advs.201500211)
Supplement: Supplementary file 1 — Supplementary [file ADVS-3-0l-s001.pdf]

Copyright WILEY-VCH Verlag GmbH & Co. KGaA, 69469 Weinheim, Germany, 2015.

ADVANCED SCIENCE
Open Access

Supporting Information

for *Adv. Sci.*, DOI: 10.1002/advs.201500211

A Two-Photon Excitation Based Fluorogenic Probe for Sialome Imaging in Living Systems

*Lei Rong, Chi Zhang, Qi Lei, Si-Yong Qin, Jun Feng, and Xian-Zheng Zhang**

Copyright WILEY-VCH Verlag GmbH & Co. KGaA, 69469 Weinheim, Germany, 2013.

Supporting Information

A two-photon excitation based fluorogenic probe for glycoprotein imaging in living systems

*Lei Rong, Chi Zhang, Qi Lei, Si-Yong Qin, Jun Feng, and Xian-Zheng Zhang**

Chemical and cell culturing materials

The following chemicals were used as received without further purification:

4-Bromo-1,8-naphthalic anhydride, D-mannosamine hydrochloride, (trimethylsilyl)acetylene, Dichlorobis(triphenylphosphine)-palladium, copper(I) iodide (CuI), tetrabutylammonium fluoride (1M in THF), and sodium azide were purchased from Sigma-Aldrich (USA).

Peracetylated N-azidoacetylmannosamine (Ac4ManNAz) was acquired from Thermo Fisher Scientific (USA).

Other chemicals used were provided by Shanghai Chemical Co. (China).

Dulbecco's Modified Eagle Medium (DMEM) cell culture media, foetal bovine serum (FBS), and phosphate buffered saline (PBS, pH= 7.4) were purchased from Life Technologies Corporation (USA), and used as recommended.

Propidium iodide (PI) were purchased from Sigma-Aldrich Co. LLC. (USA).

Trypsin-EDTA solution (0.05%, with phenol red) and Penicillin-Streptomycin solution (10,000 U/mL) were purchased from Biological Industries Israel Beit-Haemek Ltd. (Israel), and used as recommended.

Instrumentation

NMR

^{1}H NMR spectra were recorded on a Varian Unity 300 MHz spectrometer. 1H chemical shifts values, 300 MHz, are reported as δ using the residual solvent signal as internal standard.

Fluorescence spectra

Fluorescence spectra were acquired by RF-5301PC spectrofluorophotometer. (SHIMADZU, Japan.)

Fluorescence microscopy

Fluorescence microscopy images were taken using a Carl Zeiss fluorescence microscopy, NOL-LSM 710, with two-photon excitation imaging unit.

Flow cytometry

Flow cytometry data were collected by a BD Biosciences multicolor flow cytometry, BD FACSAria™ III.

Synthesis of Naph-yne

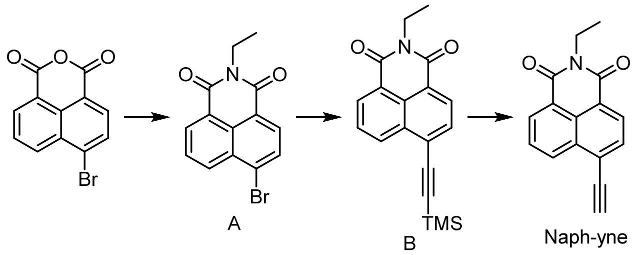

The Naph-yne was synthesized using the same protocol as we used in our previous work.[1]

Synthesis of compound A

To a solution of 4-bromo-1,8-naphthalic anhydride in DMF, excessive ethylamine were added, and the reaction mixture was heated to 100 °C and stirred for about 8 h. After cooling to room temperature, the solvent was removed under vacuum to give the compound A as brown solid. Yield: quantitative.

Synthesis of compound B

Dichlorobis(triphenylphosphine)-palladium (0.10 mmol), CuI (0.10 mmol), dry triethylamine (4.5 mmol) and (trimethylsilyl)acetylene (3.0 mmol) were added to a solution of compound A (2.0 mmol) in dry DMF under argon atmosphere. The reaction mixture was

degassed by three frezze-thraw cycles and stirred at 30 °C for 36 h. After that, the solvent was removed under reduced pressure. The crude product was purified by flash column chromatography on silica gel to obtain compound B as brown solid. Yield: 37 %.

Synthesis of Naph-yne

Tetrabutylammonium fluoride (2 mL 1M in THF, 2.0 mmol) was added to a solution of compound B (0.7 mmol) in THF. The mixture was stirred at 25 °C for about 14 h. After that, the reaction mixture was poured into water and extracted with ethyl acetate. The organic phase was washed with brine (3 x 25 mL) and dried over anhydrous $MgSO_4$. The solvent was then removed under reduced pressure. The crude product was purified by flash column chromatography on silica gel to obtain product Naph-yne as a yellow solid. Yield: 70 %. ^1H NMR (DMSO-d6, 300 MHz): 1.22 (3H, t, J 7.1 Hz, CH_3), 4.07 (1H, s, C≡CH), 5.10 (2H, q, J 7.1 Hz, CH_2), 7.96 (1H, dd, J 7.5 Hz, J 8.2 Hz, Ar-CH-7), 8.03 (1H, d, J 7.6 Hz, Ar-CH-4), 8.44 (1H, d, J 7.6 Hz, Ar-CH-3), 8.57 (1H, d, Ar-CH-6), 8.64 (1H, d, Ar-CH-8).

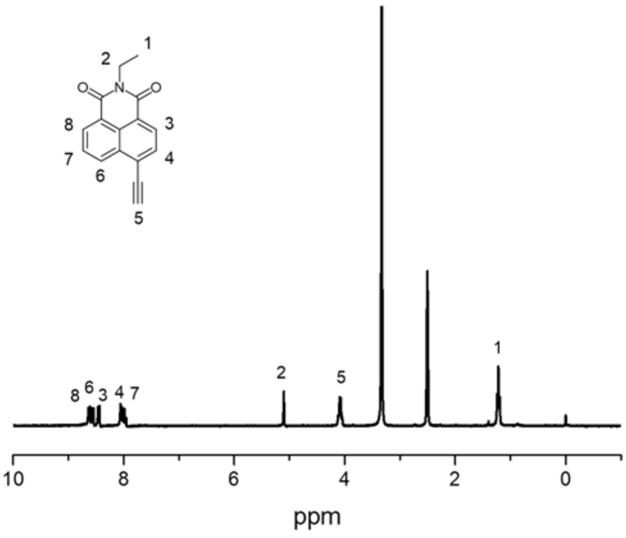

Fluorescence quantum yield measuring

The fluorescence quantum yield of Naph-yne was measured by comparing the integrated fluorescence intensities and the absorbency values of the Naph-yne with reference sample.[2,3] Quinine sulphate in 0.1 M H_2SO_4 aqueous solution was used as standard. The fluorescence quantum yield was calculated using the following equation:

$$Q_x = Q_r(\frac{I_x}{I_r})(\frac{A_r}{A_x})(\frac{{n_x}^2}{{n_r}^2})$$

where, Q is the quantum yield of the solution, I is the measured integrated emission intensity, A is the detector response, and n is the refractive index of the solution. The x and r subscripts refer to the Naph-yne and the quinine sulphate solution, respectively. In order to minimise re-absorption effects the optical densities in the 20 mm fluorescence cuvette were kept under 0.1 at the excitation wavelength.

Optimized reaction time determination

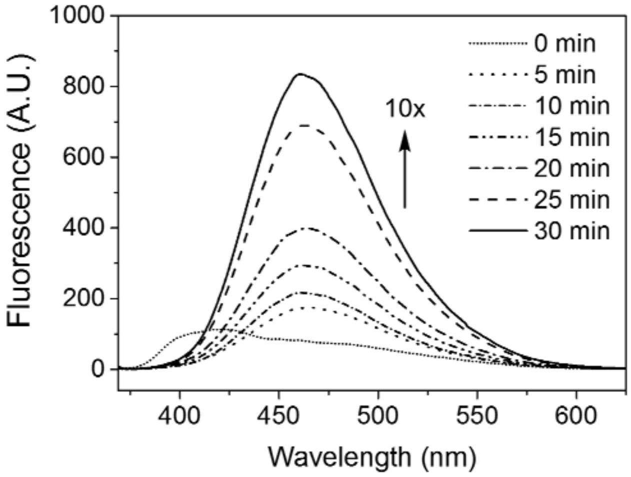

Fig. S1 The fluorescence spectra of Naph-yne (5 μM) and its CuAAC reaction adducts at different reaction times under simulated physiological conditions (PBS pH 7.4, 37 °C). The fluorescence intensity of the solution after CuAAC reaction increased sharply within 30 min compared with that of Naph-yne (0 min), and the fluorescence intensity at 30 min could fulfil the imaging needs. In consideration of the cell compatibility, the reaction time used in the subsequent experiments was 30 min.

References

[1] L. Rong, L.-H. Liu, S. Chen, H, Cheng, C.-S. Chen, Z.-Y. Li, S.-Y. Qin and X.-Z. Zhang, *Chem. Commun.* **2014**, *50*, 667.

[2] G. A. Crosby and J. N. Demas, *J. Phys. Chem.* **1971**, *75*, 991.

[3] S.-L. Hu, K.-Y. Niu, J. Sun, J. Yang, N.-Q. Zhao and X.-W. Du, *J. Mater. Chem.* **2009**, *19*, 484.
